# Fatal Neonatal Intoxication From Cutaneous Cade Oil: A Case of Multivisceral Failure

**DOI:** 10.7759/cureus.65133

**Published:** 2024-07-22

**Authors:** Salhi Chaïmae, Mohammed Ech-Chebab, Anass Ayyad, Sahar Messaoudi, Rim Amrani

**Affiliations:** 1 Department of Neonatology and Intensive Care Unit, Mohammed VI University Hospital of Oujda, Faculty of Medicine and Pharmacy of Oujda, University Mohammed First of Oujda, Maternal Child and Mental Health Research Laboratory of Oujda, Oujda, MAR

**Keywords:** cade oil, newborns, poisoning, phenol, juniper oil

## Abstract

*Juniperus oxycedrus* is a plant whose branches and wood are used to extract cade oil. This oil is widely used in traditional Moroccan medicine for its analgesic, digestive, bronchopulmonary, and dermatological properties. However, it contains toxic phenols like guaiacol and cresol, which can cause serious side effects across various organ systems, including renal, hepatic, cardiac, pulmonary, neurological, gastrointestinal, dermatological, hematological, and metabolic.

We report the case of a newborn hospitalized in neonatal intensive care at Mohammed VI University Hospital in Oujda, Morocco, following cutaneous exposure to cade oil. The newborn was admitted with acute cardiovascular shock, rapidly progressing to multiorgan failure. Despite intensive resuscitation measures, the patient died on the second day of hospitalization.

## Introduction

Cade oil is a product extracted from the branches and wood of *Juniperus oxycedrus* [1]. It is known as "qtran rqeq" oil in the Moroccan dialect [2]. It is commonly used in traditional Moroccan medicine for its analgesic effects, digestive and bronchopulmonary properties, and dermatological benefits. However, while multiorgan toxicity affecting multiple organ systems is well documented in adults, there are few reported cases in pediatric patients, especially neonates [3]. These manifestations can lead to serious or even fatal poisoning, particularly in infants and children [3,4].

We document the case of a newborn admitted to the neonatal intensive unit at Mohammed VI University Hospital in Oujda, Morocco, who had been exposed to cade oil through the skin and was admitted in a rapidly evolving state of shock, progressing to multiorgan failure. Despite intensive resuscitation efforts, the outcome was unsuccessful.

## Case presentation

A 12-day-old newborn, born from pregnancy with minimal prenatal monitoring and delivered at full term via medicalized vaginal delivery, was admitted for management of generalized hypotonia. The mother reported using cade oil as an abdominal massage oil to alleviate the infant's colic during the day, approximately eight hours before the evaluation.

**Figure 1:**
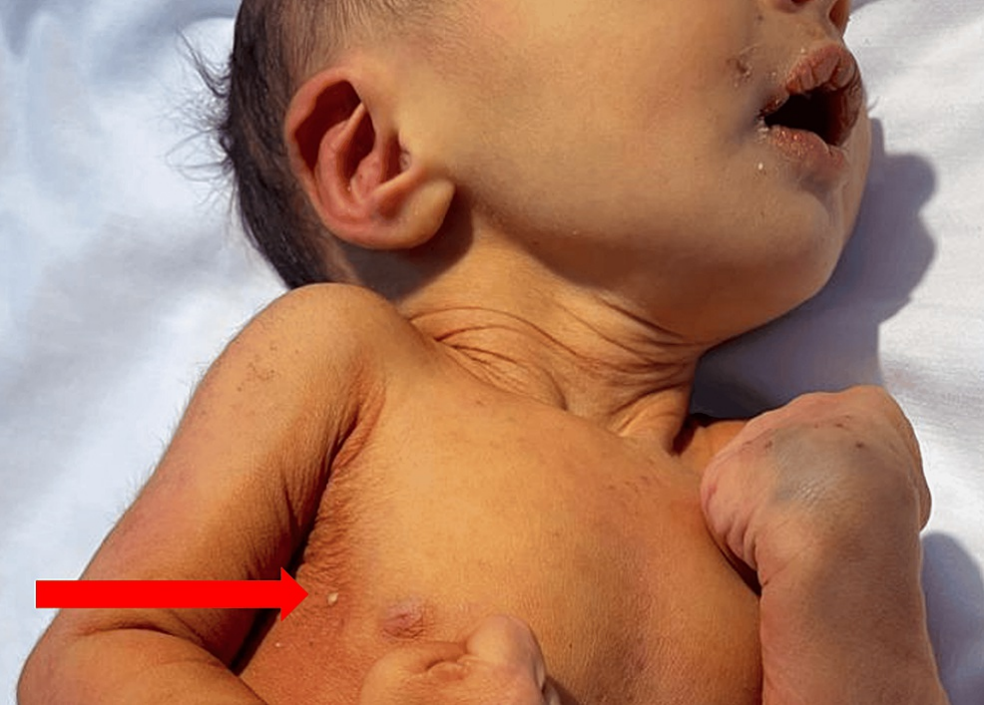
Patient showing pustular lesions on the trunk indicated by the red arrow

Upon clinical examination and admission to the neonatal intensive care unit, the newborn emitted a distinct odor compatible with cade oil. He was icteric and hypotonic, with weak archaic reflexes. He showed signs of dehydration, respiratory distress with a Silverman score of 3/10, and hemodynamic shock characterized by tachycardia at 193 beats per minute, surpassing the normal range for neonates of 130 ± 45 beats per minute, along with unmeasurable blood pressure. The temperature was 37°C, and the capillary blood glucose was 0.89 g/L. He also presented pustular lesions on the trunk (Figure 1). The remainder of the clinical examination was unremarkable.

The laboratory work-up revealed renal failure, cytolysis, hepatic cholestasis, hyponatremia, and metabolic acidosis. The rest of the laboratory workup was normal (Table 1). Toxicological tests for phenol and methemoglobinemia were not performed, as they were unavailable. The chest X-ray was unremarkable.

**Table 1:** Patient's laboratory results on admission SGOT: serum glutamic oxaloacetic transaminase, SGPT: serum glutamic pyruvic transaminases, LDH: lactate dehydrogenase, CPK: creatine phosphokinase

Laboratory parameters	Values	Reference range
Urea g/L	13.50 g/L	2.4-10.6 g/L
Creatinine mg/L	21.3 mg/L	2.4-8.5 mg/L
SGOT UI/L	119 UI/L	5-43 UI/L
SGPT UI/L	164 UI/L	0-55 UI/L
LDH UI/L	740 UI/L	125-243 UI/L
CPK U/L	857 UI/L	100-400 UI/L
Total bilirubin mg/L	182 mg/L	2-12 mg/L
Direct bilirubin mg/L	123 mg/L	0-5 mg/L
Sodium mEq/l	119 mEq/l	133-146 mEq/L
Alkaline reserve mEq/L	7 mEq/L	22-28 mEq/L
pH	7	7.35-7.45
Bicarbonate level mmol/L	20 mmol/L	22-28 mmol/L

The newborn was admitted to the neonatal intensive care unit, where he underwent skin decontamination with soapy water. Subsequently, mechanical ventilation was initiated to manage hemodynamic and acid-base imbalances. Unfortunately, the newborn's condition deteriorated, culminating in multiorgan failure and death on the second hospital day.

## Discussion

In our Moroccan context, the growing use of medicinal plants, often perceived as natural and safe treatments, raises significant concerns about potentially fatal poisoning risks. This underscores the country's substantial public health challenges [5]. Cade oil, or oxidized cedar tar oil, also known as "qtran rqeq," plays a significant role in medicinal practices in Morocco, being obtained by dry distillation of the branches of *Juniperus oxycedrus*, belonging to the Cupressaceae family [1,2]. This essential oil contains various components such as etheric oils, triterpenes (grape cadinene), and phenols (derived from guaiacol and cresol), used for many purposes in Moroccan folk and traditional medicine [3,4].

Cade oil is widely used in human and veterinary dermatology for its keratolytic, antipruritic, and antimicrobial activities [1]. Available to consumers without a prescription in Morocco, it is applied locally in folk medicine to treat psoriasis, eczema, and scabies and is used as an antiparasitic and antiseptic [4]. It is present in various cosmetic products (soap, cream, shampoo, etc.) and is also administered orally as a deworming agent [3]. Effects such as reduced blood pressure and anti-inflammatory properties have also been observed, reinforcing its traditional use [6,7]. However, cade oil presents a significant risk of intoxication, mainly attributable to phenol, which is responsible for the majority of systemic symptoms observed. The rapid absorption of phenol and its predominant hepatic metabolism lead to the formation of semi-quinone radicals during hydroxylation, which, in excess of the hepatic conjugation capacity, can oxidize and produce toxic free radicals [8].

Clinical manifestations identified in the literature following dermal or rectal exposure, ingestion, or inhalation of cade oil are well-documented in adults and in a in a few cases in children, especially neonates [7]. Acute renal failure is considered one of the main side effects of phenol, representing an unfavorable prognosis [3]. It is often accompanied by acute tubular necrosis, secondary to hemodynamic disturbances and precipitation of hemoglobin or myoglobin in the renal tubules [1,4]. Liver damage is characterized by hepatic cytolysis with centrilobular necrosis, leading to elevated transaminases, lactate dehydrogenase, and creatine kinase, although these values usually return to normal within a few months [3,4]. Cardiac damage includes severe hypotension, which can rapidly progress to cardiovascular shock, as well as tachycardias and dysrhythmias [7,9]. In addition, heart failure can develop, accompanied by elevated cardiac enzymes [1]. Pulmonary complications often begin with symptoms of respiratory distress and dyspnea, which can progress to pneumonia [1]. In more severe cases, acute pulmonary edema has also been reported, particularly in infants [7]. Damage to the central and peripheral nervous systems has been reported as involuntary movements, convulsions, dizziness, headache, hypotonia, and myoclonic coma [9]. Gastrointestinal involvement, with abdominal pain and mild gastrointestinal erosive lesions, has been reported [1,7]. Acute pancreatitis is also a rare complication of this intoxication [6]. On a dermatological level, patients often present with urticaria, edema, maculopapular, and pustular lesions, as demonstrated by our patient [4]. Hematologic abnormalities include coagulation disorders such as methemoglobinemia, deep vein thrombosis, hemolytic anemia, leukopenia, and neutropenia [1]. Finally, metabolic disorders are manifested by electrolyte imbalances, such as metabolic acidosis, hyponatremia, hyperglycemia, and hypokalemia [1]. Our patient also had acidosis and hypernatremia.

The management of cade oil poisoning involves hospitalizing symptomatic patients in an intensive care unit for continuous monitoring and rapid intervention [1]. In cases of intoxication through skin contact, washing all contaminated areas with soapy water is crucial to limit cutaneous absorption and the progression to systemic intoxication. Polyethylene glycol is also utilized for its ability to form insoluble complexes with phenol, thereby reducing its dermal absorption [6]. For the latter, treatment is symptomatic and focuses on correcting hemodynamic and acid-base disorders through intravenous hydration and urine alkalinization. In the event of severe respiratory distress, mechanical ventilation is initiated [6]. Hemodialysis does not effectively remove phenol and is only indicated in cases of anuric renal failure [7]. To prevent liver damage caused by the accumulation of free radicals, some poison control centers and authors recommend the use of N-acetylcysteine (NAC) in the treatment of intoxication by essential oils (such as pennyroyal oil and clove oil), the phenols present in cade oil, and hepatotoxic plants [7,10]. NAC helps replenish glutathione levels, is a source of essential sulfhydryl groups, and acts as a free-radical scavenger thanks to its antioxidant properties [7]. The administration of methylene blue is indicated for the management of methemoglobinemia [6]. By integrating these specific strategies into management, it is possible to significantly improve the prognosis of patients suffering from severe intoxication.

## Conclusions

Cade oil poisoning in infants is a critical situation that must be promptly recognized and managed appropriately. Its widespread use in Moroccan traditional medicine can expose children to serious risks of systemic toxicity. Clinical manifestations such as renal failure, hepatic cytolysis, and severe respiratory disorders underscore the importance of raising awareness among healthcare professionals and the public about the potential dangers associated with this oil. Additional research is needed to better understand its toxic effects and optimize its therapeutic use to ensure patient safety.
